# Renal angiomyolipoma presenting with massive retroperitoneal haemorrhage due to deranged clotting factors: a case report

**DOI:** 10.1186/1757-1626-1-213

**Published:** 2008-10-04

**Authors:** Timothy Wright, Prasanna Sooriakumaran

**Affiliations:** 1 Department of Urology, Royal Surrey County Hospital, Egerton Road, Guildford, Surrey, GU2 7XX, UK; 2 Department of Urology, Kingston Hospital, Galsworthy Road, Kingston, Surrey, KT2 7QB, UK

## Abstract

**Background:**

Angiomyolipomata of the kidney are unusual lesions composed of abnormal vasculature, smooth muscle, and adipose elements. They may be associated with tuberous sclerosis and occasionally present with flank pain, a palpable mass, and gross haematuria. As angiomyolipomata grow their risk of bleeding increases, with a greater than 50% chance of significant bleeding in lesions > 4 cm; anticoagulant therapy accentuates this risk.

**Case presentation:**

A case of massive retroperitoneal haemorrhage in a patient on warfarin is presented. The underlying diagnosis of renal angiomyolipoma was diagnosed based on CT findings. Emergency resuscitation and selective interpolar arterial embolization was performed which saved the patient's life as well as his kidney.

**Conclusion:**

This case illustrates the clinical scenario of massive retroperitoneal haemorrhage in an anticoagulated patient with renal angiomyolipomata. In the emergent situation, adequate resuscitation along ABC principles, as well as control of haemorrhage with either nephrectomy (partial or radical), non-selective renal arterial embolization, or selective embolization of the feeding vessel(s), is necessary. For this to occur, it is imperative to consider the diagnosis early in warfarinized patients (and others at risk of bleeding) who present with abdominal pain. The authors hope this case report highlights to readers the clinical scenario of massive retroperitoneal haemorrhage in anticoagulated patients with renal angiomyolipomata so that they can deal appropriately with such presentations.

## Background

Renal angiomyolipoma have an incidence of 0.3–3% and arise from the mesenchymal elements of the kidney [1]. It is estimated that ten million people worldwide have such lesions. They are mostly benign tumours composed of varying proportions of mature adipose tissue, smooth muscle and abnormal thick-walled blood vessels. In 5% of the tumours, fatty elements can be detected only at microscopy [2].

Angiomyolipomata have been described as both hamartomas and christomas. The actual definition though must not be that of a hamartoma as fat and smooth muscle are not normal constituents of the renal parenchyma. Rather an angiomyolipoma is a christoma as it is composed of various amounts of vascular, muscular and fatty tissues.

Angiomyolipomata may occur as an isolated phenomenon or as part of the syndrome associated with tuberous sclerosis. The overall female:male predominance is approximately 4:1 with this strong female prevalence suggesting a hormonal component to tumour growth [2]. Isolated angiomyolipomas occur sporadically, account for 80% of angiomyolipomas, are usually solitary and occur almost exclusively in women in the fourth to fifth decade of life (mean age 43 [3]). Angiomyolipomata associated with tuberous sclerosis are typically larger than isolated angiomyolipomata, have multifocal or bilateral disease, tend to occur in younger patients (third decade), and require careful screening for the presence of renal tumours. With these tuberous sclerosis associated angiomyolipomata the male:female distribution is nearly equal; however women outnumber men in terms of prevalence.

Most small angiomyolipomata are asymptomatic and found incidentally on imaging studies, usually incidental from US or CT scans performed for unrelated clinical indications. The images generally show a highly or mixed echogenic renal tumour without calcification that is difficult to distinguish from renal cell carcinoma on US and thus requires further imaging with CT. On CT, angiomyolipomata have well defined margins, with a variable proportion of fat and soft tissue, although the former usually predominates. They can vary in size from a few millimeters to larger than 20 cm. Research has shown that 25–50% of patients have some or all of the stigmata of tuberous sclerosis, which include epilepsy, mental retardation and adenoma sebaceum [4,5].

In the minority of patients that are symptomatic, they classically present with flank pain (53%), a palpable tender mass (47%) and gross haematuria (23%); this is known as 'Lenk's triad' [6]. Other symptoms associated but noted less frequently include nausea or vomiting, fever, anaemia and blood pressure changes [7]. Several studies have demonstrated that the frequency of symptoms and risk of bleeding increases with the size of the angiomyolipoma. There is a 13% risk of bleeding if the lesion is < 4 cm and a 51% risk if > 4 cm [2,7]. Most patients with small tumours (< 4 cm) that tend to be asymptomatic are managed conservatively, with annual CTs [8]. Asymptomatic lesions > 4 cm should have CT follow-up every six months and symptomatic patients generally have tumours of > 8 cm. It is these larger tumours that are at greater risk of spontaneous or traumatic rupture resulting in haemorrhagic complications. These patients are therefore treated with angiography with selective transarterial embolization as a first line. Nephrectomy, partial or radical, is indicated if there is persistent haemorrhage, suspicion of malignancy, or failed embolisation. Prophylactic embolisation of asymptomatic lesions 4 cm or larger is recommended in select high risk patients, including younger women who intend future pregnancy or in patients in whom regular follow-up is difficult [2].

The importance of this follow up has been demonstrated with documented evidence that 27% of lesions smaller than 4 cm and 46% of lesions > 4 cm have been shown to grow up to 4 cm per year over a mean follow up period of 4 yrs [1]. In all cases though, the aim of treatment is to preserve renal function and to prevent haemorrhage.

## Case presentation

A 57-year-old gentleman, presents to the accident and emergency department with acute onset, severe, colicky left loin pain radiating to the anterior chest with associated shortness of breath. Two months previously he was investigated for chest pain and was found to be in atrial fibrillation. He was commenced on warfarin and amiodarone while awaiting cardioversion.

On examination following pain relief, the patient was comfortable, afebrile and had stable observations (BP 120/80, pulse 60 irregularly irregular, oxygen saturations 97% on room air). There was however marked tenderness of the left renal angle and left lower quadrant but no signs of peritonism. Bloods (Hb 16.1), blood gases and urine dipstick performed at the time were all entirely normal and the patient was found to have an INR of 2.8 (in keeping with treatment for AF); however of note earlier in the week the INR had been reported as 5. An IVU showed no filling defects and therefore CT imaging was performed.

**Figure 1:**
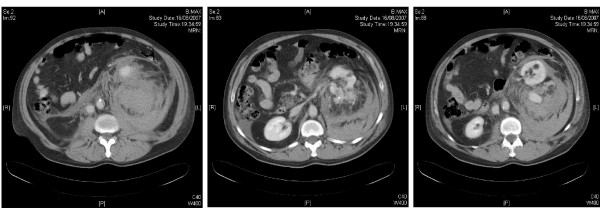
CT scans to illustrate angiomyolipomata with a massive retroperitoneal haemorrhage.

The CT showed a massive retroperitoneal bleed behind the left kidney and thus was difficult to interpret; the radiologist initially reported the bleed as arising from the psoas. Despite the use of FFP and Vitamin K his Hb continued to drop over 24 hrs from 16.1 to 5.9. Repeat examination of the CT revealed multiple angiomyolipomata found in the left kidney with the bleed originating from a sub-capsular angiomyolipoma (see Figure 1). The patient was transfused and underwent selective interpolar arterial embolisation of the left kidney. At embolization it was found that there was a large psuedoaneurysm arising from the interpolar artery as well as the abnormal vessels consistent with the vessels supplying the angiomyolipoma. Post-treatment contrast injection into the left renal artery confirmed adequate embolization and the patient was stable enough to be discharged one week later with no further evidence of bleeding.

## Conclusion

This case has presented the scenario where anticoagulation therapy is the main cause for massive haemorrhage in a patient with an angiomyolipoma. This then raises the question whether screening for angiomyolipoma should be considered in patients prior to starting warfarin therapy? There are no studies that have investigated this question, and given the large number of patients on anticoagulant therapy and the relatively small number who suffer retroperitoneal haemorhage as a result of a coexisting angiomyolipoma, it might be more sensible to simply consider the diagnosis early in warfarinized patients who present with abdominal pain.

## Abbreviations

AF: atrial fibrillation; CT: computed tomography; FFP: fresh frozen plasma; Hb: haemoglobin; INR: international normalized ratio; IVU: intravenous urogram; US: ultrasound.

## Competing interests

The authors declare that they have no competing interests.

## Authors' contributions

TW and PS were involved in the clinical care of the patient. PS conceived of the case report, TW wrote the manuscript under the guidance of PS, and PS revised it. Both authors read and approved the final manuscript.

## Consent

Written informed consent was obtained from the patient for publication of this case report and any accompanying images. A copy of the written consent is available for review by the Editor-in-Chief of this journal.
